# The Medical Relevance of *Toxoplasma* Infections in Terms of the Safety of Blood Recipients under Immunosuppression—A Meta-Analysis

**DOI:** 10.3390/microorganisms11081980

**Published:** 2023-08-01

**Authors:** Roland Wesołowski, Marta Pawłowska, Celestyna Mila-Kierzenkowska

**Affiliations:** Department of Medical Biology and Biochemistry, Faculty of Medicine, Ludwik Rydygier Collegium Medicum in Bydgoszcz, Nicolaus Copernicus University in Toruń, 87-100 Toruń, Poland; roland@cm.umk.pl (R.W.); marta.pawlowska@cm.umk.pl (M.P.)

**Keywords:** toxoplasmosis, blood donors, blood recipients, blood transfusion, diagnostics, *Toxoplasma gondii*

## Abstract

Laboratory diagnosis of *Toxoplasma gondii* infection plays a crucial role in ensuring the safety of blood recipients, especially in the case of immunosuppressed people, such as organ transplant patients. Toxoplasmosis, caused by the parasite *Toxoplasma gondii*, is a potential threat to people with weakened immune systems, and blood transfusions from infected donors can lead to severe complications. In this publication, we analyze the medical relevance of *Toxoplasma* infection in the context of the safety of blood recipients, focusing on the immunosuppressed patient population. We present various diagnostic methods, such as serological, molecular, and microscopic tests, which can detect the presence of *Toxoplasma gondii* in donors’ blood. We also discuss the importance of adequately interpreting diagnostic results, considering risk factors, and detectability of the infection. We pay special attention to high-sensitivity and -specificity diagnostic techniques, which allow us to minimize the risk of *Toxoplasma gondii* transmission to blood recipients. Our findings have important implications for clinical practice and organ transplantation guidelines, emphasizing the need to diagnose and monitor *Toxoplasma* infections in blood donors and recipients.

## 1. Introduction

Toxoplasmosis is a disease caused by an obligatory intracellular blood protozoan known as *Toxoplasma gondii*. The parasite was first discovered by Nicolle and Manceaux in 1908 in a small North African rodent called *Ctenodactylus gundi* [1]. *T. gondii* is widespread and infects a significant proportion of the global population [2,3]. The infection is usually harmless and resolves independently [4]. However, individuals with weakened immune systems are at risk of developing severe toxoplasmosis. In transplant recipients, toxoplasmosis can occur due to various factors, such as infection transmitted from the donor, reactivation of a dormant condition in the recipient, or, rarely, a new acute infection. Both solid organ transplant recipients and those who undergo hematopoietic stem cell transplantation are vulnerable to severe illness caused by *Toxoplasma*. The disease can manifest in various ways, including pneumonitis, meningoencephalitis, chorioretinitis, myocarditis, or disseminated toxoplasmosis affecting multiple organs [5]. The parasite can be transmitted directly through the donated organ or blood transfusions. Additionally, toxoplasmosis can result from the reactivation of preexisting infections or new infections acquired after the transplant. Several factors contribute to the increasing significance of parasitic infections in transplantation. The acknowledgment of parasitic infections as a significant risk to transplant outcomes has been influenced by shifts in immigration patterns and the growth of transplant medicine in developing nations. Changing global immigration patterns allows individuals from regions where parasites are endemic to undergo transplantation. Moreover, these immigrants may also serve as organ donors in developed countries [6].

Traditionally, blood screening services have primarily emphasized the detection of viruses like human immunodeficiency virus (HIV), hepatitis B and C viruses, and bacteria such as *Treponema pallidum*. However, it is crucial to expand this focus to include other significant pathogens like *Toxoplasma gondii*, which have been associated with transfusion-transmitted infections [7]. There is a high risk of acute *Toxoplasma* infection if the necessary measures are not taken to protect organ transplantation and blood transfusion patients from infection. This risk is mainly present in organ transplants or blood transfusions from seropositive donors to seronegative recipients [8]. The risk of transfusion transmission of *T. gondii* is particularly elevated in individuals who receive multiple blood transfusions, neonates, pregnant women, and patients under immunosuppression. These groups have a reduced capacity to mount an effective immune response against the parasites, making them more susceptible to infection [3,7,9]. The focus on HIV and transfusion-transmitted hepatitis infections has overshadowed the recognition that other diseases, specifically parasites such as *T. gondii*, can also be transmitted through blood transfusions, resulting in severe conditions, particularly among immunocompromised patients [7]. Blood donors with positive immunoglobulin M (IgM) serum can often pose a risk factor for susceptible recipients [10]. Thus, it is essential to acknowledge the potential impact of this parasite and take appropriate measures to prevent its transmission during transfusion procedures.

The purpose of this paper is to highlight the significance of *Toxoplasma gondii* as a pathogen in blood transfusion and transplantation settings and emphasize the need to expand blood screening services to include the detection of this parasite. This paper aims to raise awareness about the risks of *T. gondii* infection in individuals with weakened immune systems, particularly blood transfusion recipients and organ transplant recipients under immunosuppression. It also addresses the potential transmission of *T. gondii* through blood transfusions or donated organs and the factors contributing to the increasing importance of parasitic infections in transplantation.

## 2. Methods

We conducted an extensive literature search using the ISI Web of Science/PubMed/Science Direct/Google Scholar databases for information on the risk of toxoplasmosis in blood recipients. The following keywords were used in data retrieval: (“toxoplasmosis” and “blood donors”, “blood recipients”, “transfusion”, “transplant recipients”); (“*Toxoplasma gondii*”, and “blood donors”, “blood recipients”, “transfusion”, “transplant recipients”); (“toxoplasmosis diagnostics” and “blood donors”, “blood recipients”, “transfusion”, “transplant recipients”). There were no restrictions in collecting the data. Conference abstracts and studies with incomplete or unavailable data were excluded.

## 3. Results

### 3.1. The Classical Course of Toxoplasma Infection and Toxoplasmosis

Toxoplasmosis is significant as a zoonotic disease. *T. gondii* can infect approximately one-third of the world’s population [7]. There are several routes of *T. gondii* infection in humans. These include ingesting food or water contaminated with oocysts shed by cats, which can be found in soil or garbage [11]. Consumption of undercooked or raw meat containing tissue cysts is also a potential source of infection [1,2]. Additionally, *T. gondii* can be transmitted through open wounds, transplantation, blood transfusion, congenital transmission from infected mothers to their babies, and even sexual contact [7]. In exceptional circumstances, seropositive blood donors, especially those in the acute phase of infection, may potentially contribute to transmission [12]. The transmission of *T. gondii* through blood product transfusion remains a rather unexplored area, posing a current challenge for transfusion medicine [13].

*T. gondii* has developed a sophisticated strategy to proficiently manipulate the host’s immune system, establishing productive infection and ensuring optimal replication [14]. The life cycle of *T. gondii* exhibits complexity, involving felids as definitive hosts and warm-blooded vertebrates, specifically mammals and birds, as intermediate hosts. This intricate life cycle encompasses various stages of sexual and asexual reproduction [15]. In humans, two forms of *T. gondii* are observed. The actively proliferating tachyzoites are typically seen during the infection’s initial and more acute phase. The slowly dividing bradyzoites, on the other hand, form cysts in the brain and skeletal muscles due to the host’s immune response [7]. The parasite infiltrates human tissues and cells upon infection, which undergoes multiplication through internal budding, known as endodyogeny. During the acute stage of *Toxoplasma* infection, the parasites replicate within host cells and form pseudocysts. These asexual stages of *T. gondii* give rise to merozoites, which enter the bloodstream and establish cysts in various tissues [7].

Approximately 80% to 90% of individuals with a healthy immune system, including children and adults, experience asymptomatic infection. Among those who exhibit symptoms, the most prevalent clinical presentation is the presence of isolated cervical or occipital lymphadenopathy, occasionally accompanied by a mild, transient influenza-like illness [16,17]. Toxoplasmosis can give rise to severe and potentially life-threatening manifestations in individuals with compromised immune systems [14]. Such embodiments may include acute symptoms like encephalitis and pneumonia, primarily stemming from the reactivation of latent infection [17,18].

In the case of primary infection, serological tests can identify three distinct sequences: the emergence of IgM, the rise in IgG levels, and the subsequent stabilization of IgG, potentially accompanied by the persistence of IgM. Conversely, reactivation is characterized by two sequences: an elevation in IgG levels followed by their stabilization. IgG antibodies remain detectable throughout a person’s lifetime in residual titers, indicating the risk of reactivation during immunosuppression [2]. 

### 3.2. Prevalence of Toxoplasma Infections in Blood Donors

The *Toxoplasma* seropositivity can differ widely among different world regions (10–80%) and sometimes within a country, depending on social and cultural habits and transmission routes [12,19]. Many epidemiological studies screened blood donors for *Toxoplasma* infection. Results of some studies, presented in Table 1, provide insights into the prevalence and co-occurrence of IgG and IgM anti-*T. gondii* antibodies, as well as the presence of *T. gondii* DNA in healthy blood donors. Alvarado-Esquivel et al. [20] conducted a study investigating the relationship between age and seropositivity. Their research findings revealed an increase in the frequency of seropositivity with advancing age. Furthermore, the authors observed a negative correlation between seropositivity to *T. gondii* and the educational level of the donors, indicating a decrease in seroprevalence as the educational attainment of the individuals increased. Hosseini et al. [19] studied the presence of antibodies against *Toxoplasma gondii* and the presence of parasite DNA among Iranian blood donors. The results showed that 73.5% of the blood donors tested positive for anti-*T. gondii* IgG antibodies, indicating previous exposure to the parasite, while 2.2% were positive for IgM antibodies, suggesting recent infection. *T. gondii* DNA was detected in 7 (of 400) samples. The genotyping analysis revealed the presence of four *T. gondii* genotypes (ToxoDB#1, #2, #10, and #27), with 50% of the strains being highly pathogenic. Rodrigues et al. [21] conducted a study to assess the seroprevalence of exposure to or infection with *T. gondii* among blood donors who underwent three blood tests at the Portuguese Institute for Blood and Transplantation banks, as well as various regional Blood Collection Services in Portugal. The findings revealed a seroprevalence rate of 38.1% among the participants, indicating a significant level of exposure to or infection with *T. gondii*. Nearly half of the individuals who tested positive for *T. gondii* had no prior knowledge of *Toxoplasma* infection. However, it is worth noting that anti-*Toxoplasma* antibodies persist long and do not necessarily mean an acute infection [22]. In turn, Pawełczyk et al. [23], in a group of 168 blood donors, observed 1 (0.6%) IgM-positive and 49 (29.2%) IgG-positive subjects. Further research showed that 15 (8.9%) IgM-negative and IgG-negative blood donors had *T. gondii* DNA present. Thus, *T. gondii* DNA detection in seronegative subjects implies supplementing routine serological testing via molecular methods. 

The burden of *T. gondii* infection among blood donors remains poorly documented due to limited information. In Poland, the examination of blood donors is regulated based on the regulation of the Ministry of Health regarding the conditions for collecting blood from blood donors. This regulation specifies that blood samples from qualified individuals require the following markers: HBs antigen, anti-HIV 1/2 antibodies, anti-HCV antibodies, HCV RNA, HBV DNA, HIV RNA, and syphilis treponemal infection markers [24]. Blood donors are, therefore, not routinely screened for *Toxoplasma* infection. Therefore, the risk of transfusion transmission of pathogen may be undetected because the donor might test negative in serological tests during the active phase of *T. gondii* infection.

Regrettably, screening for this parasite in blood banks is not mandatory worldwide [10]. The parasite has demonstrated the ability to remain viable for up to 50 days in blood or its components, even when stored at 4 °C. Additionally, it can survive in citrated blood at 5 °C for up to 50 days, including the buffy coat, thereby raising the possibility of acquiring the infection through blood, platelets, or leukocyte transfusions [10,25,26,27]. Consequently, preserving blood bags within the cooling chain does not prevent transmission or delay the infection [10]. 

Given this circumstance, it is crucial to actively disseminate information about *Toxoplasma* infection and promote preventive measures among the general population, including individuals who participate as blood donors. Such efforts are imperative to raise awareness and adopt appropriate preventive practices. Many studies suggest enhancing blood safety measures of pregnant, immunocompromised, and multi-transfused patients. For this purpose, the screening for *T. gondii* as a priority test for all pretransfusion blood testing schedules should be performed (especially in regions with high endemicity) [26,28,29,30,31,32].

**Table 1:** Summary of selected research studies on *Toxoplasma* infection status in healthy blood donors.

Size of the Study Group	Analyzed Parameter	Method	Results	Refs.
*n* = 790	IgG and IgM anti-*T. gondii* antibodies	ECLIA	213 (24.2%)–only IgG (+) 8 (0.9%)–only IgM (+) 12 (1.4%)–both IgM (+) and IgG (+)	[8]
*n* = 385	IgG and IgM anti-*T. gondii* antibodies DNA of *T. gondii*	ELISA, LAMP	146 (37.9%)–only IgG (+) 4 (1.03%)–only IgM (+) 6 (1.56%)–both IgM (+) and IgG (+) 6 (1.56%)–*T. gondii* DNA (+)	[33]
*n* = 1347	IgG and/or IgM anti-*T. gondii* antibodies	LAT	618 (45.9%)–IgM (+) and/or IgG (+)	[26]
*n* = 375	IgG and IgM anti-*T. gondii* antibodies	ELISA	94 (25.1%)–only IgG (+)	[34]
*n* = 800	IgG and IgM anti-*T. gondii* antibodies	IFA, ELISA	352 (44%)–only IgG (+) 3 (0.4%)–both IgM (+) and IgG (+)	[12]
*n* = 103	IgG and IgM anti-*T. gondii* antibodies	ELISA	46 (47.7%)–only IgG (+)	[28]
*n* = 462	IgG and IgM anti-*T. gondii* antibodies DNA of *T. gondii*	ELISA, PCR (529 bp)	150 (32.5%)–only IgG (+) 7 (1.5%)–only IgM (+) 9 (2%)–both IgM (+) and IgG (+) 30 (18%) of 166 seropositive donors–*T. gondii* DNA (+)	[10]
*n* = 400	IgG and IgM anti-*T. gondii* antibodies DNA of *T. gondii*	ELISA, nested-PCR multilocus, nested-PCR-RFLP	294 (73.5%)–only IgG (+) 9 (2.2%)–only IgM (+) 7 (1.8%)–both IgM (+) and IgG (+) 7 (1.8%)–*T. gondii* DNA (+)	[19]
*n* = 46	IgG and IgM anti-*T. gondii* antibodies	ELISA	11 (23.9%)–only IgG (+) 1 (2.2%)–only IgM (+)	[35]
*n* = 380	IgG and IgM anti-*T. gondii* antibodies	ELISA	131 (34.47%)–only IgG (+) 2 (0.5%)–only IgM (+) 11 (2.9%)–both IgM (+) and IgG (+)	[36]
*n* = 520	IgG anti-*T. gondii* antibodies	MAT	198 (38.1%)–IgG (+)	[21]
*n* = 510	IgG and IgM anti-*T. gondii* antibodies DNA of *T. gondii*	ELC, nested-PCR, qPCR	223 (43.7%)–only IgG (+) 8 (1.6%)–both IgM (+) and IgG (+) all samples–*T. gondii* DNA (−)	[37]
*n* = 750	IgG and IgM anti-*T. gondii* antibodies DNA of *T. gondii*	ELISA, nested-PCR (*B1* gene)	335 (44.7%)–only IgG (+) 5 (0.6%)–only IgM (+) 21 (2.8%)–both IgM (+) and IgG (+) 38 (10.8%) of IgG (+)–*T. gondii* DNA (+)	[13]
*n* = 150	IgG anti-*T. gondii* antibodies DNA of *T. gondii*	ELISA, real-time PCR (*B1* gene)	98 (65.3%)–only IgG (+) 15 (10%)–*T. gondii* DNA (+)	[38]
*n* = 1783	IgG and IgM anti-*T. gondii* antibodies DNA of *T. gondii*	ELISA, real-time PCR	161 (9.3%)–only IgG (+) 5 (0.28%)–both IgM (+) and IgG (+) all samples–*T. gondii* DNA (−)	[39]
*n* = 207	IgM anti-*T. gondii* antibodies	ELISA	46 (22.2%)–IgM (+)	[30]
*n* = 500	IgG and IgM anti-*T. gondii* antibodies DNA of *T. gondii*	ELISA, real-time PCR	144 (28.8%)–only IgG (+) 11 (2.2%)–only IgM (+) 5 (1%)–both IgM (+) and IgG (+) 1 (9%) of IgM (+)–*T. gondii* DNA (+)	[31]
*n* = 194	IgG and IgM anti-*T. gondii* antibodies	CLIA	75 (38.66%)–only IgG (+) 2 (1.03%)–only IgM (+)	[32]
*n* = 1480	IgG and IgM anti-*T. gondii* antibodies DNA of *T. gondii*	ELISA, nested-PCR (*B1* gene)	182 (12.3%)–only IgG (+) 81 (5.47%)–only IgM (+) 23 (1.6%)–both IgM (+) and IgG (+) 2 (1.9%) of IgM (+)–*T. gondii* DNA (+)	[40]

(+) positive; (−) negative; CLIA, chemiluminescence immunoassay; ECLIA, electrochemiluminescence immunoassay; ELC, electrochemiluminescence; ELISA, enzyme-linked immunosorbent assay; IFA, indirect fluorescent antibody; LAMP, loop-mediated isothermal amplification; LAT, latex agglutination test; MAT, modified agglutination test; PCR, polymerase chain reaction.

### 3.3. Prevalence of Toxoplasma Infections in Blood and Transplant Recipients

**Figure 1:**
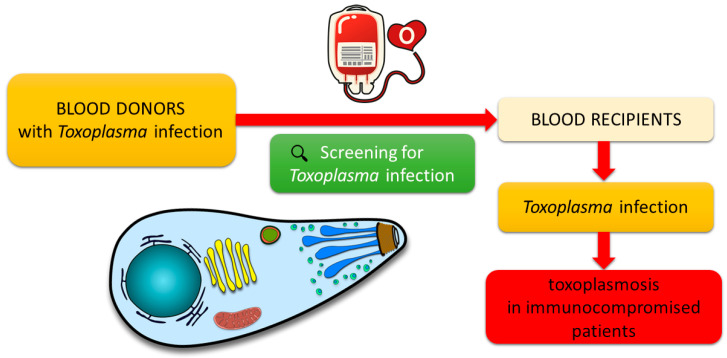
Blood transfusion as a possible source of *Toxoplasma gondii* infection.

*Toxoplasma gondii* can be transmitted to transplant recipients, including those undergoing heart, renal, and bone marrow transplantation, and through blood products. Patients who are organ recipients use immunosuppressive drugs that lower their immunity. Organ recipients often also require a blood transfusion, so they are also blood recipients. They are, therefore, a group of patients particularly vulnerable to toxoplasmosis. Hence, particularly in this group, special measures must be taken to prevent acute toxoplasmosis and its serious effects. Consequently, screening healthy blood donors for the presence of *T. gondii* is crucial (see Figure 1) [3,19]. It is known that donating blood from seropositive donors to individuals under immunosuppression or seronegative recipients for organ transplantation may cause *Toxoplasma* contamination. This can result in severe health complications for the affected patients [8]. 

Mardani [22] emphasizes that the widespread utilization of blood and blood components and the occurrence of asymptomatic toxoplasmosis in blood donors due to the absence of effective diagnostic strategies render the donor selection strategy inadequate in preventing transfusion-transmitted parasitic infections. For parasitic agents to be transmitted via transfusion, several conditions must be met: (1) they must be present in the bloodstream of donors either for a prolonged duration or in a sufficient quantity to pose a risk to susceptible recipients; (2) they should be capable of causing infection in the absence of clinical symptoms; (3) they must be able to survive during the storage period of blood and its components; and (4) they should have a relatively lengthy incubation period [22]. Belkacemi and Heddi [28] indicate that the potential risk of *T. gondii* transmission through blood donations ranges from 1 in 100,000 to 17 in 100,000. Siegel et al. [41] reported that four individuals with acute leukemia developed toxoplasmosis after receiving leukocyte transfusions from donors with chronic myelogenous leukemia. A retrospective analysis of serologic data from the donors revealed that their anti-*Toxoplasma* antibody levels were significantly elevated. This finding suggests that the transferred leukocytes originated the *Toxoplasma* parasite in the recipients [41].

Transfusion-acquired toxoplasmosis is the primary cause of ocular *Toxoplasma* infection, which occurs when the chronic proliferation of tachyzoites in the retina or hypersensitivity response to ruptured tissue cysts occurs. Ocular manifestations of toxoplasmosis, such as uveitis, can occur in individuals with conditions like thalassemia, requiring frequent and regular blood transfusions from multiple donors for survival. Consequently, asymptomatic blood donors infected with *T. gondii* may unknowingly transmit the parasite during blood transfusion [7]. Toxoplasmosis in recipients of cord blood transplantation often arises from the reactivation of a chronic infection, the transmission of disease from the donor, or a newly acquired infection. The risk of developing the condition has been associated with organ transplants such as heart, liver, kidney, and stem cell transplants [25]. The prevalence of *T. gondii* infection among recipients of hematopoietic stem-cell transplantation ranges from 0.4% to 9% and is strongly associated with significant immunosuppression [4]. Cerebral toxoplasmosis is the most common clinical manifestation of the infection in immunocompromised patients, followed by the progression of pulmonary disease leading to acute respiratory distress syndrome and disseminated toxoplasmosis. However, due to the non-specific nature of the symptoms, a definitive diagnosis of *Toxoplasma* infection is sometimes only established through a post-mortem necropsy [4].

### 3.4. Laboratory Diagnosis of Toxoplasma Infections in Terms of the Safety of Blood and Transplant Recipients

The transmission of *T. gondii* from seemingly healthy blood donors to recipients has emerged as a significant concern in transfusion medicine, particularly among recipients with compromised immune systems. Screening methods for *T. gondii* infection in blood donors primarily rely on serological tests, such as the Sabin-Feldman test, indirect hemagglutination test, direct hemagglutination, indirect immunofluorescence test, and enzyme-linked immunosorbent assay for detecting anti-*Toxoplasma* IgG/IgM antibodies in serum [7]. However, serological diagnosis can be challenging in immunocompromised patients, as the reactivation of *T. gondii* or the chronic phase of infection may not lead to noticeable changes or detectable shifts in antibody levels. Interpreting serology results in immunocompromised transplant recipients can be challenging, particularly in non-specific febrile illnesses. This is especially true for individuals with AIDS and certain chronic cases of *Toxoplasma* infection, where IgM antibodies may persist, complicating the interpretation of serological results. Additionally, molecular screening, immunoblotting, and tissue biopsy have been utilized to identify active *T. gondii* infections [7]. Several real-time PCR assays and PCR targets have been created to rapidly and effectively detect *T. gondii*. One of these targets is the *B1* gene, which is present in 35 copies in the parasite’s genome. Recently, researchers have identified another repeat element in *T. gondii*, which appears around 300 times in the genome and spans 529 base pairs [42]. The 529 bp repeat element has been extensively studied and explored as a PCR detection target [3]. This element shows promise as a target for real-time PCR, potentially leading to improved performance compared to the *B1* gene as a target. According to Edvinsson et al. [42], low concentrations of *T. gondii* DNA can be detected more sensitively and accurately by real-time PCR using the 529 bp repeat element of *T. gondii* than when using the *B1* gene. Quantitative real-time PCR targeting the 529 bp repeat region has demonstrated the highest efficacy among various PCR-based diagnostic approaches, successfully validated in several significant indications [2]. Thus, molecular screening for *Toxoplasma* infection is recommended before blood transfusion, particularly in immunocompromised patients [25]. Polymerase chain reaction (PCR) testing can aid in expediting the diagnosis, especially for patients at higher risk due to discordant donor/recipient *Toxoplasma* IgG status and inability to tolerate TMP/SMX prophylaxis [6]. 

Screening donors for *Toxoplasma* IgG is now a requirement under the policy of the Organ Procurement and Transplantation Network/United Network for Organ Sharing (UNOS/OPTN) [6]. Diagnosing acute *Toxoplasma* infection in solid organ transplant (SOT) recipients is recommended to utilize PCR testing on blood and various bodily fluids and perform biopsies on affected tissues to identify tachyzoites. Currently, therapy for toxoplasmosis in solid organ transplantation recipients includes an introduction and chronic suppressive therapy (based on pyrimethamine, sulfadiazine, and leucovorin) [6]. However, these recommendations do not refer to the diagnosis and use of prophylaxis for blood transfusion recipients. Therefore, it seems reasonable to assess the current health status of blood donors and recipients and to analyze the safety of blood transfusions regarding the risk of *Toxoplasma* infection. 

Wang et al. [43] indicate that Chinese blood centers typically employ leukocyte filtration, effectively lowering the presence of obligatory intracellular pathogens, including *T. gondii*. Consequently, the risk of *T. gondii* infection is significantly diminished. Therefore, there is no need to conduct *T. gondii* screening for blood donations in China. However, there are no investigations of the patients who received *T. gondii* DNA-positive blood. Therefore, the risk of the possibility of transfusion transmission of *T. gondii* is not determined [43]. The lack of research implies that it would be reasonable to include the screening of blood donors for *Toxoplasma* infections in the event of a need to perform a transfusion in organ recipients.

Obtaining *T. gondii* isolates from blood donors or donated samples would be beneficial. However, this poses challenges due to the short duration of parasitemia, which is limited to the acute phase of infection. Isolating viable parasites from blood samples is challenging. Alternatively, detecting the parasite’s mRNA in donated blood could be considered, but this procedure may interfere with routine processes in blood banks and contaminate the blood bags [13]. These difficulties contribute to the limitations of studies that have explored molecular approaches to detect *T. gondii* infection among blood donors. Molecular methods such as conventional and nested PCR cannot differentiate between alive and dead parasites and residual DNA. These methods can only indicate the transmission risk and may overestimate the presence of the parasite in peripheral blood [44].

Due to the high prevalence of *T. gondii* infection among blood donors and the absence of an approved and dependable laboratory test for *Toxoplasma* screening in donors, it is not feasible to prevent transfusion transmission of the pathogen solely through donor selection and serological screening methods. Additionally, discarding blood donations based on positive serology test outcomes poses a significant risk to blood availability, particularly in countries with a high infection prevalence [22].

## 4. Conclusions

Problem: This paper highlights the potential risk of *Toxoplasma gondii* transmission through blood transfusion, particularly in vulnerable populations such as immunocompromised individuals, organ transplant recipients, neonates, and pregnant women.

Solution: To effectively manage *Toxoplasma gondii* infections in transplant recipients and reduce the risk of transmission, it is crucial to update recommendations regarding the screening of blood donors for the presence of the parasite. Comprehensive donor testing and implementation of preventive measures are essential in protecting patients from acute *Toxoplasma* infection during transfusion.

Concrete results: This paper provides valuable insights into the importance of screening blood donors, improving transfusion management, and developing *Toxoplasma* infection prevention programs. These measures can significantly enhance patient safety and reduce the likelihood of parasite transmission through blood transfusion.

Future challenges: Despite increased awareness, there are still gaps in knowledge concerning the specific strategies and protocols needed for managing *Toxoplasma gondii* infections in vulnerable populations. Further research is necessary to better understand the burden of *T. gondii* infection among blood donors and to develop more comprehensive screening and preventive strategies.

In conclusion, by implementing updated screening procedures and adopting appropriate strategies, we can minimize the risk of acquiring *Toxoplasma* transmission through blood transfusion and ensure the well-being of susceptible patient groups. Continued efforts in research and collaboration are essential to further enhance our understanding and management of *Toxoplasma* infections in the context of blood transfusion.

## Data Availability

The data presented in this study are available on request from the corresponding author. The data are not publicly available due to privacy or ethical restrictions.
